# Impact of colonization with multidrug-resistant organisms on antibiotic prophylaxis in patients with cirrhosis and variceal bleeding

**DOI:** 10.1371/journal.pone.0268638

**Published:** 2022-05-24

**Authors:** Victoria T. Mücke, Kai-Henrik- Peiffer, Johanna Kessel, Katharina M. Schwarzkopf, Jörg Bojunga, Stefan Zeuzem, Fabian Finkelmeier, Marcus M. Mücke

**Affiliations:** 1 Department of Internal Medicine 1, University Hospital Frankfurt, Goethe University, Frankfurt am Main, Germany; 2 Department of Internal Medicine 2, University Hospital Frankfurt, Goethe University, Frankfurt am Main, Germany; Medizinische Fakultat der RWTH Aachen, GERMANY

## Abstract

**Background:**

The efficacy of antibiotic prophylaxis to prevent rebleeding or infection after variceal bleeding in patients with liver cirrhosis colonized with multidrug-resistant organisms (MDROs) is unknown.

**Methods:**

In this retrospective study, patients with liver cirrhosis and endoscopically confirmed variceal bleeding who were treated at a tertiary care center in Germany and were screened for MDROs at the time of bleeding were eligible for inclusion. Efficacy of antibiotic prophylaxis was evaluated in patients stratified according to microbiological susceptibility testing.

**Results:**

From 97 patients, the majority had decompensated liver cirrhosis (median MELD Score 17) and ACLF was present in half of the patients (47.4%). One third of patients were colonized with MDRO at baseline. De-novo infection until day 10 or the combination of de-novo infection or rebleeding were comparable among both groups (p = 0.696 and p = 0.928, log-rank-test). Risk of de-novo infection or rebleeding was not significantly increased in patients who received antibiotic prophylaxis that did not cover the MDRO found upon baseline screening. Acute-on-chronic liver failure at baseline was the strongest and only independent risk factor that was associated with both outcomes (OR 5.52, 95%-CI 1.48–20.61, p = 0.011 and OR 11.5, 95%-CI 2.70–48.62, p<0.001). Neither MDRO colonization at baseline nor covering all detected MDRO with antibiotic prophylaxis (i.e. “adequate” prophylaxis) impacted transplant-free survival. Again, the presence of ACLF was the strongest independent risk factor associated with mortality (OR 9.85, 95%-CI 3.58–27.12, p<0.0001).

**Conclusion:**

In this study, MDRO colonization did not increase the risk of rebleeding, infections nor death, even if antibiotic prophylaxis administered did not cover all MDRO detected at MDRO screening. Patients with ACLF had an increased risk of bleeding, infections and death.

## Introduction

Variceal bleeding in patients with cirrhosis and portal hypertension is one of the most severe and instantly life-threatening complication [1]. Then, immediate therapy with vasoactive agents (e.g. terlipressin) and early endoscopic treatment is warranted. Bacterial infections are reported in most patients with acute variceal haemorrhage and are often suspected to be present upon admission. They may be the initial cause of decompensation and often precipitate the bleeding event itself [2].

Antibiotic prophylaxis has become the standard of care in patients with cirrhosis and acute variceal bleeding [3, 4], as randomized controlled trials have shown a significant benefit with regards to infection control and survival [5].

However, these studies date back 20–30 years ago. Since then, the number of infections with gram-positive bacteria as well as with multidrug-resistant organisms (MDROs) are increasingly observed in these patients [6–8]. Lately, rates of MDRO colonization in patients with cirrhosis have been reported to be up to 40–50% increasing to about 75% in a collective on long-term antibiotic prophylaxis to prevent spontaneous bacterial peritonitis (SBP) [9, 10]. Prado et al. recently showed that patients colonized with MDRO often develop infections caused by the colonizing bacteria during follow-up [10]. Accordingly, efficacy of long-term antibiotic prophylaxis to prevent SBP has been shown to have become less effective over the last decades in a recent meta-analysis [9] and reduced efficacy was linked to MDRO colonization in one prospective observational study [11].

So far, effects of MDRO colonization on short-term antibiotic prophylaxis in patients with cirrhosis and variceal bleeding have not been investigated. In fact, in most studies that have been conducted, patients were not screened for MDRO colonization and patients were often excluded if they previously received antibiotic therapy, a common risk factor for MDRO development [5, 8]. Apart from that, a recent analysis of more than 2,000 patients with acute variceal bleeding reported that bacterial infection develops in one fifth of patients despite antibiotic prophylaxis [12].

Thus, aim of this study was to assess the impact of MDRO colonization on the efficacy of antibiotic prophylaxis in patients with endoscopically confirmed variceal bleeding.

## Materials and methods

### Study design

All patients with liver cirrhosis who were admitted to the Department of Internal Medicine, University Hospital Frankfurt, Germany from June 2010 to June 2021 were eligible for inclusion. The patient database of the endoscopy department of the University Hospital Frankfurt was retrospectively and systematically searched. Cases were included if patient had liver cirrhosis and endoscopically confirmed signs of upper gastrointestinal variceal bleeding. All patients had to have undergone MDRO screening within 24 hours of bleeding diagnosis. The diagnosis of liver cirrhosis was based on histology from liver biopsy (if available) or by the combination of clinical, imaging and laboratory findings. Acute-on-chronic liver failure (ACLF) was diagnosed according to standard criteria [13]. In recurring patients, first bleeding episode was chosen as index bleeding episode.

Patients were excluded if they were younger than 18 years old, were pregnant, had received solid organ transplantation before, were under immunosuppressive therapy or had any malignancy other the hepatocellular carcinoma within Milan criteria. The local ethics committee approved this study (vote 20–707).

### Clinical data collection

Information obtained from systematically reviewed charts were summarized in a data collection form. It included patients’ characteristics such as gender, age, aetiology of cirrhosis, past medical history, laboratory values and stage of liver cirrhosis, including model for end-stage liver disease (MELD), CLIF-C-ACLF-score upon diagnosis, endoscopic findings, medical and endoscopic treatments, including antibiotic prophylaxis, risk factors for MDRO development, MDRO screening results upon diagnosis, development of ACLF during hospital stay, development of (MDRO) infections, de-novo colonization or rebleeding within the next 3 months, and liver transplantation or death.

### Microbiological monitoring

Upon intensive care unit admission patients received routine MDRO screenings within 24 hours of the bleeding event via nasal/oral and rectal swabs for study inclusion. Microbiological culture procedures and antibiotic susceptibility testing are described elsewhere [11]. A bacterial isolate was considered to be an MDRO if it had an acquired non-susceptibility to at least one in three or more antimicrobial categories: extended-spectrum beta-lactamase (ESBL, *Escherichia coli* and *Klebsiella pneumoniae*), *carbapenem-resistant Acinetobacter baumanni and Pseudomonas spp*. as well as *Stenotrophomonas maltophilia*, *Achromobacter xylosidans*, an equivalent of derepressed chromosomic AmpC ß-lactamase-producing *Enterobacteriaceae* (*Enterobacter spp)*, *vancomycin-resistant Enterococcus spp*. *(VRE) and methicillin resistant staphylococcus aureus (MRSA)*.

### Aim of the study and definition of outcomes

In this study we aimed to investigate the impact of MDRO colonization on the efficacy of recommended antibiotic prophylaxis following upper gastrointestinal variceal bleeding. Efficacy of prophylaxis was measured as de-novo infection and rebleeding within 10 days. A second analysis to assess antibiotic efficacy was performed to avoid possible bias in the MDRO colonization group. For this scenario patients were divided in those with “adequate” and those who might be considered with “inadequate” prophylaxis. Antibiotic prophylaxis was considered adequate if 3^rd^ generation cephalosporins were used in patients without MDRO colonization upon admission. Other broad-spectrum antibiotics could be used for prophylaxis in patients with suspected infection or MDRO colonization. Then prophylaxis was considered adequate if the antibiotics covered the MDRO that was found upon screening (i.e. carbapenems in patients with an ESBL Enterobacteriaceae). In this scenario, antibiotic prophylaxis was considered inadequate if the MDRO detected was not covered by the antibiotics used (i.e. 3^rd^ generation cephalosporins in patients with an ESBL Enterobacteriaceae in rectal swaps was considered inadequate). Secondary outcome was transplant-free survival.

### Statistical analysis

For statistical analysis, BiAS, Version 11.03 was used. Group differences were assessed by the Mann-Whitney-U-Test and Fisher’s exact test for continuous or categorical variables, respectively. For outcomes time-to-events were estimated with Kaplan-Meier methods and differences were compared with the Logrank test.

Univariate and multivariate logistic-regression analysis was performed to analyze factors associated with de-novo infection or rebleeding within 10 days and transplant-free survival after one year after bleeding event using backward selection and a *P* value ≥ 0.10 for removal from the model. Only patients with complete data sets for the remaining covariates were included in regression analyses. Odds ratios (ORs) and respective 95% confidence intervals (CIs) were calculated for each variable. Two-sided *P* values < 0.05 were considered to be statistically significant.

## Results

### Patients’ characteristics

Overall, 97 patients (median age 57 years, interquartile-rage IQR 10 years) with endoscopically confirmed variceal bleeding (VB) and valid MDRO screening without HCC outside Milan could be included. Within the timeframe, the database of the endoscopy department comprised 606 encoded incidences of variceal bleeding and liver cirrhosis. All duplicates were reduced to the first bleeding episode as the index case (n = 437 duplicates removed). Then we excluded underaged patients (n = 2), patients without liver cirrhosis with hindsight (n = 12), cases with insufficient documentation (n = 9), patients with carcinoma other than HCC inside MILAN (n = 44), patients with intraoperative bleeding (n = 3) and two cases without variceal bleeding, but otherwise coded. Detailed patients’ characteristics and laboratory values can be found Table 1. Seventy-six patients (78.4%) were male, half of the them had alcoholic liver cirrhosis, 24 (24.7%) had liver cirrhosis due to chronic viral hepatitis. The majority of patients had decompensated liver cirrhosis (Child-Pugh B/C n = 82 (84.5%), median MELD-Score 17 with an interquartile range (IQR) of 5, ascites was present in 71 patients (73.2%) and in 75 patients (77.3%) previous variceal state was known. Thirty-six patients (37.1%) had received previous endoscopic variceal therapy, 4 patients (4.1%) transjugular intravenous portosystemic shunt (TIPS) placement. The majority of patients presented with hematemesis (n = 68, 70.1%) and or melena (n = 89, 91.8%) and ACLF was present at the time of bleeding in 46 patients (47.4%) with a median CLIF-C ACLF score of 59 (IQR 8). The median time to endoscopy from diagnosis was 2 hours and 66 patients (68.0%) had active bleeding upon endoscopy. Almost all patients received endoscopic treatment (n = 91, 93.8%) and antibiotic prophylaxis (n = 94, 96.6%), all patients received terlipressin, but only 77 patients (79.4%) for 3–5 days. 3^rd^ generation cephalosporines as recommended in current guidelines was the antibiotic prophylaxis of choice (n = 59, 60.8%), but also other antibiotic regimens were administered, either due to presence of MDRO or in case of additional suspected infections, including carbapenems (n = 21, 21.6%) and/or additional glycopeptides (n = 33, 34.0%).

**Table 1:** Patients’ characteristic for patients stratified according to baseline MDRO colonization.

Characteristics	All patients (n = 97)	MDRO colonization (n = 34)	No MDRO colonization (n = 63)	P-value
Age, y, median (IQR)	57 (10)	54 (8)	58 (10)	0.372
Male sex, n (%)	76 (78.4)	25 (73.5)	51 (81.0)	0.444
Etiology of cirrhosis				
Alcohol, n (%)	48 (49.5)	21 (61.8)	27 (42.9)	0.091
Viral Hepatitis, n (%)	24 (24.7)	7 (20.6)	17 (27.0)	0.802
NASH, n (%)	4 (4.1)	1 (2.9)	3 (4.7)	1.000
Cryptogenic, n (%)	5 (5.2)	1 (2.9)	4 (6.3)	0.654
other, n (%)	16 (16.5)	4 (11.8)	12 (19.0)	0.407
ICU admission during hospital stay, n (%)	93 (95.9)	33 (97.1)	60 (95.2)	1.000
Days on ICU, median (IQR)	4 (2)	4 (1)	3 (1)	0.180
Hemorrhagic shock at bleeding, n (%)	54 (55.7)	20 (58.8)	34 (54.0)	0.674
Stage of liver disease				
MELD-Score, median (IQR)	17 (5)	18 (8)	17 (5)	0.991
Child-Pugh B/C n (%)	82 (84.5)	28 (82.4)	54 (85.7)	0.770
Ascites, n (%)	71 (73.2)	24 (70.6)	47 (74.6)	0.811
ACLF present at bleeding, n (%)	46 (47.4)	19 (55.9)	27 (42.9)	0.287
Risk factors for MDRO				
Prior hospitalization, n (%)	69 (71.1)	24 (70.6)	45 (71.4)	1.000
Prior ICU admission, n (%)	24 (24.7)	8 (23.5)	16 (25.4)	1.000
Prior systemic antibiotics, n (%)	41 (42.3)	21 (61.8)	20 (31.7)	**0.005**
Prior MDRO infections, n (%)	2 (2.1)	2 (5.9)	0 (0)	0.121
Laboratory results, median (IQR)				
C-reactive protein (mg/dl)	1.1 (0.7)	1.4 (1.0)	1.1 (0.7)	0.465
White blood count (/nl)	8.2 (2.0)	8.4 (1.6)	7.8 (1.9)	0.463
Hemoglobin (g/dl)	7.9 (1.4)	7.5 (1.0)	8.3 (1.9)	0.352
Serum Sodium (mmol/l)	138 (5)	138 (3)	137 (5)	0.565
Bilirubin (mg/dl)	2.1 (1.0)	1.5 (0.5)	2.3 (1)	0.290
Creatinine (mg/dl)	1.1 (0.4)	1.2 (0.4)	1.1 (0.4)	0.505
International normalized ratio	1.5 (0.2)	1.6 (0.3)	1.6 (0.2)	0.721
Albumin (g/dl)	2.7 (0.5)	2.7 (0.7)	2.7 (0.4)	0.457
Platelets (/nl)	82 (25)	86 (21)	78 (21)	0.368
Endoscopy findings/therapy				
Time to endoscopy, hours, IQR	2 (1)	2 (1)	2 (1)	1.000
Grade of EV	2 (0)	2 (0)	2 (0)	1.000
Active bleeding at endoscopy	66 (68.0)	25 (73.5)	41 (65.5)	0.500
Additional fundus varices	31 (32.0)	9 (26.5)	22 (34.9)	0.500
Endoscopic treatment				
EV ligature	71 (73.2)	26 (76.5)	45 (71.4)	0.639
Injection therapy	19 (19.6)	7 (20.6)	12 (19.0)	1.000
EV Stenting	3 (3.1)	1 (2.9)	2 (3.2)	1.000
TIPS placement during hospitalization	11 (11.3)	4 (11.8)	7 (11.1)	1.000
Terlipressin treatment for 3–5 days	77 (79.4)	30 (88.2)	47 (74.6)	0.187
Outcome, n (%)				
ACLF day 7	26 (26.8)	12 (35.3)	14 (22.2)	0.230
Liver transplantation	6 (6.2)	1 (2.9)	5 (7.9)	0.662
Death/liver transplantation within				
30 days, n (%)	28 (28.9)	12 (35.3)	16 (25.4)	0.345
365 days, n (%)	39 (40.2)	15 (44.1)	24 (38.1)	0.665

Abbreviations: NASH, non-alcoholic steatosis hepatis; ICU, intensive care unit; IQR, interquartile range; MELD, model end stage liver disease; ACLF, acute-on-chronic liver failure; MDRO, multidrug resistant organism; EV, esophagus varices; TIPS, transjugular intravenous portosystemic shunt.

Patients were followed for a median of 148 days (IQR 132 days). Transplant-free survival was 68.2% after 30 days and 39.1% within one year. Overall, 27 (27.8%) had a confirmed de-novo infection within 10 days. Mostly pulmonary infections were observed (29.6%), directly followed by abdominal and urinary tract infections (both 22.2%, for details see S1 Table). Twenty patients (20.6%) had a rebleeding event within 10 days. Thirteen patients (13.4%) had de-novo MDRO infection.

### MDRO colonization and efficacy of antibiotic prophylaxis

One third of patients (n = 34, 35.1%) were colonized with MDRO at baseline, ESBL E. coli being the most prevalent bacteria (n = 16,16.5%). In 31 patients (32.0%) de-novo MDRO colonization was detected within 3 months with a median time to de-novo colonization of 12 days (IQR 6 days). Probably due to the use of 3^rd^ generation cephalosporines, VR Enterococci were the most prevalent bacteria detected in screenings during follow-up (n = 22, 22.7%). Detailed information on MDRO colonization at baseline and during follow-up, and on MDRO infections can be found in S2 Table.

**Fig 1:**
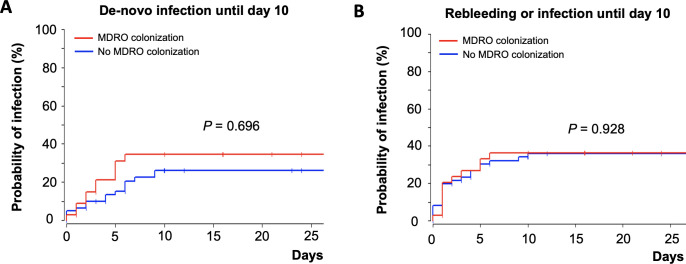
Kaplan-Meier curve depicting time-to event analysis in patients with and without MDRO colonization at baseline with regards to de-novo infection until day 10 (A) and the composite outcome of rebleeding or infection until day 10 (B).

Patients with and without MDRO colonization at baseline were comparable in most aspects. As expected, patients with prior systemic antibiotic therapy (last three months) were more likely to be colonized with MDRO at baseline (p = 0.005). Days of antibiotic treatment did not differ between both groups (p = 0.111). Of note, de-novo infection until day 10 (log-rank test, p = 0.696) or the combination of rebleeding or de-novo infection (log-rank test, p = 0.928) were not significantly different between patients with and without MDRO colonization at baseline (Fig 1). Development of ACLF at day 7 and transplant-free survival were comparable though numerically higher in the MDRO group.

**Fig 2:**
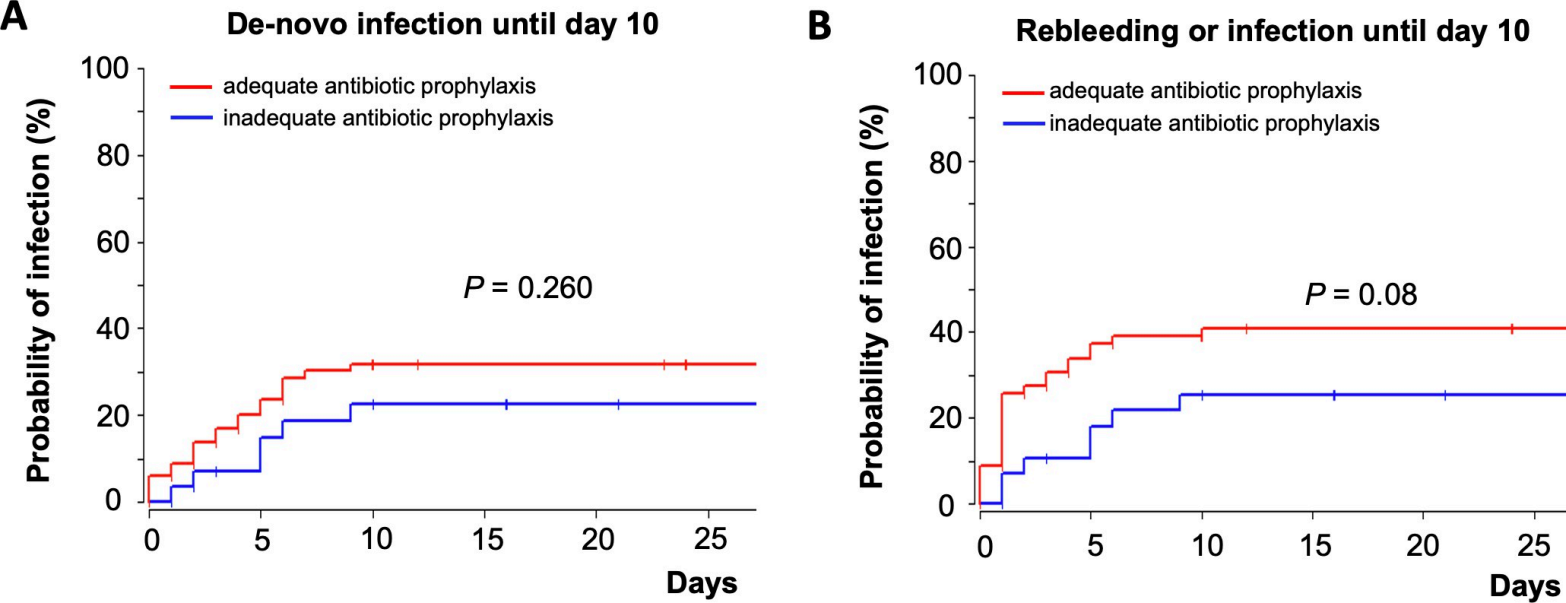
Kaplan-Meier curve depicting time-to event analysis in patients with adequate and inadequate antibiotic prophylaxis following the bleeding event with regards to de-novo infection until day 10 (A) and the composite outcome of rebleeding or infection until day 10 (B).

A second analysis to assess antibiotic efficacy was performed to avoid possible bias in the MDRO colonization group. For this scenario patients were stratified in those with an antibiotic prophylaxis that covered all MDRO that were isolated in baseline screening, the so-called “adequate antibiotic prophylaxis” group and those with an antibiotic prophylaxis that did not cover all MDRO, the so called “inadequate antibiotic prophylaxis” group (see methods section). Per definition, patients with MDRO colonization were more likely to be in the inadequate antibiotic prophylaxis group (p<0.0001), yet all other patients’ characteristics were well distributed among both groups and were statistically comparable (S3 Table). With regard to the defined outcomes, occurrence of de-novo infections within 10 days was comparable in the adequate and inadequate treated patients (log-rank test p = 0.26, Fig 2A). Risk of rebleeding or de-novo infection within 10 days was not significantly increased in the group with inadequate antibiotic prophylaxis. In fact, the risk of this compositive outcome was numerically higher in the group of patients with standard antibiotic prophylaxis without MDRO or with an antibiotic prophylaxis that covered all detected MDRO at baseline (log-rank test, p = 0.08, Fig 2B).

### Multivariate analysis: De-novo infection, rebleeding and transplant-free survival

Multivariate analyses were performed to investigate independent risk factors for infection or rebleeding within 10 days and transplant-free survival (Tables 2–4). Of note, MDRO colonization was not associated with de-novo infections or rebleeding within 10 days after the initial bleeding event. The strongest and only independent predictor of infections and rebleeding within 10 days was the presence of ACLF at baseline (OR 5.52, 95%-CI 1.48–20.61, p = 0.011 and OR 11.5, 95%-CI 2.70–48.62, p<0.001). Variceal ligature therapy was associated with reduced rebleeding risk at day 10 (OR 0.10, 95%-CI 0.02–0.46, P = 0.003). Risk of infection or rebleeding did not differ among those with 3, 4 or 5 days of antibiotic prophylaxis (OR 1.11, 95%-CI 0.61–2.00, p = 0.737 and OR 0.99, 95%-CI 0.51–1.89, p = 0.965, respectively) in a separate analysis.

**Table 2:** Uni- and multivariate logistic regression analysis investigating risk factors for de-novo infection within 10 days under antibiotic prophylaxis.

Variables	Univariate analysis		Multivariate analysis	
Model 1	OR (95% CI)	P-value	OR (95% CI)	**P-value**
Age	1.00 (0.96–1.04)	0.914		
Gender, female	0.40 (0.11–1.53)	0.182		
Days on ICU	0.94 (0.90–0.97)	0.012		
Diabetes mellitus	2.13 (0.77–5.89)	0.146		
Previous SBP	3.92 (1.06–14.48)	0.041		
MELD-Score	0.94 (0.89–0.99)	0.032		
ACLF present at bleeding	7.17 (2.36–21.74)	0.0005	5.52 (1.48–20.61)	0.011
Concomitant infection at bleeding	4.99 (1.83–13.6)	0.002		
MDRO colonization at baseline	1.28 (0.49–3.32)	0.618		
MDRO colonization during follow-up	1.42 (0.78–2.58)	0.256		

Abbreviations: ACLF, acute-on-chronic liver failure; CI, confidence interval; ICU, intensive care; MELD, model for end-stage liver disease; MDRO, multidrug-resistant organism; SBP, spontaneous bacterial peritonitis.

**Table 3:** Uni- and multivariate logistic regression analysis investigating risk factors for rebleeding within 10 days under antibiotic prophylaxis.

Variables	Univariate analysis		Multivariate analysis	
Model 1	OR (95% CI)	P-value	OR (95% CI)	P-value
Age	1.04 (1.00–1.09)	0.063	1.08 (1.02–1.14)	0.005
Gender, female	0.57 (0.15–2.22)	0.418		
Days on ICU	0.96 (0.92–1.00)	0.079		
MELD-Score	1.04 (0.93–1.17)	0.472		
ACLF present at bleeding	4.45 (1.44–13.72)	0.009	11.5 (2.70–48.62)	<0.001
Concomitant infection at bleeding	2.82 (1.01–7.89)	0.048		
MDRO colonization at baseline	0.52 (0.17–1.62)	0.262		
MDRO colonization during follow-up	1.93 (0.98–3.81)	0.058	2.57 (1.10–6.02)	0.030
Grade of varices	0.81 (0.37–1.80)	0.603		
Active bleeding at endoscopy	1.32 (0.42–4.14)	0.634		
EV ligature therapy	0.35 (0.12–1.00)	0.050	0.10 (0.02–0.46)	0.003
TIPS insertion	0.49 (0.09–2.65)	0.403		

Abbreviations: ACLF, acute-on-chronic liver failure; CI, confidence interval; EV, esophageal varices; ICU, intensive care; MELD, model for end-stage liver disease; MDRO, multidrug-resistant organism; SBP, spontaneous bacterial peritonitis; TIPS, transjugular intrahepatic portosystemic shunt.

**Table 4:** Uni- and multivariate logistic regression analysis investigating risk factors for death or liver transplantation after one year.

Variables	Univariate analysis		Multivariate analysis	
Model 1	OR (95% CI)	P-value	OR (95% CI)	P-value
Age	0.96 (0.93–1.00)	0.033	0.94 (0.89–0.99)	0.015
ACLF present at bleeding	11.57 (4.43–30.24)	<0.0001	9.85 (3.58–27.12)	<0.0001
Concomitant infection at bleeding	5.17 (2.11–12.69)	0.003		
MDRO colonization at baseline	0.98 (0.42–2.27)	0.958		
MDRO colonization during follow-up	0.85 (0.50–1.42)	0.532		
Active bleeding at endoscopy	2.52 (1.02–6.23)	0.045		
Adequate antibiotic prophylaxis	2.13 (0.86–5.32)	0.102		
TIPS insertion	0.19 (0.04–0.94)	0.041		
Infection within 10 days	3.78 (1.44–9.94)	0.007		
Rebleeding within 10 days	4.45 (1.45–13.66)	0.009	4.59 (1.12–18.83)	0.034

Abbreviations: ACLF, acute-on-chronic liver failure; CI, confidence interval; MELD, model for end-stage liver disease; MDRO, multidrug-resistant organism; TIPS, transjugular intrahepatic portosystemic shunt.

Of note, neither MDR colonization at baseline nor covering all detected MDRO with antibiotic prophylaxis (i.e. “adequate” prophylaxis) impacted transplant-free survival. Again, the presence of ACLF at baseline was the strongest independent risk factor associated with mortality after one year (OR 9.85, 95%-CI 3.58–27.12, p<0.0001). De-novo infection and rebleeding within 10 days were both associated with increased mortality in univariate analysis (OR 3.78, 95%-CI 1.44–9.94, p = 0.007 and OR 4.45, 95%-CI, p = 0.009) but only rebleeding predicted independently death in multivariate analysis (OR 4.59, 95%-CI 1.12–18.8, p = 0.034).

## Discussion

In our study, we investigated for the first time the impact of MDRO colonization on the effectiveness of short-term antibiotic prophylaxis in patients with cirrhosis and acute variceal bleeding. Here, neither early de-novo infection nor rebleeding were seen more frequently in these patients. In a second analysis in which patients’ antibiotic prophylaxis was stratified according to microbiological susceptibility testing, risk of early de-novo infection or rebleeding were comparable between those that received antibiotics that covered all MDRO detected and those who received standard prophylaxis.

Since the establishment of antibiotic stewardship programs, the usage of antibiotics in patients without confirmed or suspected infection is critically looked at. Yet, several studies have shown the benefit of antibiotic prophylaxis in patient with variceal bleeding, as it reduces the risk of rebleeding, infection and is associated with increased survival [5]. However, most studies were conducted in the 1990s, mainly used fluoroquinolones as antibiotics and rates of MDRO colonization and infection have significantly increased since then [6, 7]. Rates of MDRO colonization have been reported from 40–50% to more than 75% in some populations on long-term antibiotic prophylaxis [9, 10]. Similarly, we observed a baseline colonization with MDRO of 35%, which increased to 58% during follow-up.

There is a growing evidence that long-term, standard chinolon-based antibiotic prophylaxis to prevent SBP might be less effective in the setting of MDRO colonization [11]. Moreover, empirically use of broad-spectrum antibiotic therapy has been strongly advocated in critically ill patients with cirrhosis and suspected infection to address the increasing prevalence of MDROs [3]. This raises the question as to whether the antibiotic prophylaxis in patients with variceal bleeding should cover all known MDROs at bleeding diagnosis. If so, the use of carbapenems and even an additional use of glycopeptides as antibiotic prophylaxis would be the consequence.

In our study, MDRO colonization was frequent. Despite antibiotic prophylaxis, several de-novo infections were observed and in case of MDRO infections, almost all infections were caused by the MDRO that colonized the patients. However, we could not observe an increased risk of rebleeding or death in patients with MDRO colonization. In a second analysis, patients were grouped in those with an antibiotic prophylaxis that covered all MDRO that were isolated at baseline screening, the so-called “adequate antibiotic prophylaxis” group and those with an antibiotic prophylaxis that did not cover all MDRO, the so called “inadequate antibiotic prophylaxis” group. No benefit was seen with regard to early rebleeding or infection in the group that received antibiotics covering all detected MDROs and “inadequate prophylaxis” was not associated with increased mortality.

It has been acknowledged that the occasional acquisition of MDRO may lead to intestinal MDRO colonization and then, due to (repetitive or long-term) antibiotic exposure or other substances with an antibacterial effect, to intestinal domination of MDRO [14]. Subsequently, these MDRO can possibly be harmful to their host. Accordingly, we were able to show in a prospective trial that long-term antibiotic prophylaxis became less effective in patients with known MDRO colonization or infections [11]. Data from this study, however, suggests, that efficacy of short-term antibiotic prophylaxis remained unaffected and patients can be treated with cephalosporins regardless of the susceptibility profile of MDRO that colonized the patient.

Several studies have already demonstrated that patients with ACLF are at increased risk of (de-novo) infection or poor outcome [13, 15–17]. Similarly in our study, the only and strongest independent predictor for de-novo infections or rebleeding was the presence of ACLF. Moreover, presence of ACLF was a strong and independent predictor of mortality in our study.

Thus, broad spectrum antibiotic therapy remains justified in patients at risk, such as patients with ACLF or pre-ACLF if infection is suspected [7, 18]. However, our data suggest, others should receive standard antibiotic prophylaxis and should be closely monitored. As expected, we observed an increased selection of *Enterococci* and risk of VRE colonization following ceftriaxone-based prophylaxis, that has been earlier reported [19]. Antibiotic regimens in patients with ACLF or sepsis should, therefore, be tailored accordingly.

Limitations of our study are the retrospective, monocentric design. As a result, data is possibly prone to reporting and information bias. Patients who received previous antibiotic treatment or had suspected infections were not excluded as in earlier studies, since MDROs often develop following antibiotic therapy and infection are common among patient with variceal bleeding. As both groups (MDRO vs. no MDRO colonization and “adequate” and “inadequate” antibiotic prophylaxis) had well distributed characteristics, no matching was necessary to stratify for additional confounding factors. Yet, future prospective trials are needed to confirm our results as number of patients are limited in our study and subgroups may be underpowered (e.g. patients with TIPS). Furthermore, our study was not designed to assess if all patients still benefit from antibiotic prophylaxis or if there are subgroups that do not need antibiotic prophylaxis.

## Conclusions

In conclusion, this retrospective study was able to show that MDRO colonization does not impact standard short-term antibiotic efficacy with cephalosporins in patients with acute variceal bleeding. ACLF was the strongest and independent predictor of rebleeding, de-novo-infection or death, thus broad-spectrum antibiotic therapy may be justified in cases with suspected infection or sepsis. Future prospective trials are needed to confirm our results.

## Supporting information

S1 Table Focus of confirmed de-novo infection within 10 days after bleeding event. (PDF)

S2 Table Detailed characteristics of MDRO colonization at admission, de-novo colonization during follow-up and MDRO infection during follow-up. (PDF)

S3 Table Patients’ characteristics according to the adequacy of antibiotic prophylaxis. (PDF)

S1 File (DAT)

S2 File (DAT)

## List of abbreviations

ACLF: acute-on-chronic liver failure
CI: confidence interval
CR: carbapenem-resistant
ESBL: Extended-spectrum beta-lactamase
IQR: interquartile range
MELD: model for end-stage liver disease
MDRO: multidrug-resistant organisms
MRSA: methicillin-resistant *Staphylococcus aureus*
OR: odds ratio
SBP: spontaneous bacterial peritonitis
TIPS: transjugular intravenous portosystemic shunt
VB: variceal bleeding
VRE: vancomycin-resistant *Enterococcus spp*
