# Supplementary material for: Impact of colonization with multidrug-resistant organisms on antibiotic prophylaxis in patients with cirrhosis and variceal bleeding
Source: PLoS One. 2022 May 24;17(5):e0268638. doi: 10.1371/journal.pone.0268638 (PMC9128949; doi:10.1371/journal.pone.0268638)
Supplement: S1 Table — (PDF) [file pone.0268638.s001.pdf]

S1 Table. Focus of confirmed de-novo infection within 10 days after bleeding event.

**Focus**	**Patients with de-novo infection (n=27)**
Pulmonal	8 (29.6)
Abdominal	6 (22.2)
Urinary	6 (22.2)
Blood	3 (11.1)
Skin	3(11.1)
Other	1 (3.7)
