# Supplementary material for: Impact of colonization with multidrug-resistant organisms on antibiotic prophylaxis in patients with cirrhosis and variceal bleeding
Source: PLoS One. 2022 May 24;17(5):e0268638. doi: 10.1371/journal.pone.0268638 (PMC9128949; doi:10.1371/journal.pone.0268638)
Supplement: S2 Table — (PDF) [file pone.0268638.s002.pdf]

S2 Table. Detailed characteristics of MDRO colonization at admission, de-novo colonization during follow-up and MDRO infection during follow-up.

Characteristics	All patients (n=97)
MDRO colonization at admission, n (%)	34 (35.1)
ESBL E. coli, n (%)	16 (16.5)
ESBL K. pneumoniae, n (%)	0 (0)
CR Enterbacteriaceae, n (%)	3 (3.1)
Amp-C Enterobacter spp., n (%)	2 (2.1)
CR P. aeruginosa spp., n (%)	2 (2.1)
VR Enterococcus spp., n (%)	13 (13.4)
other MDR bacteria, n (%)	2 (2.1)
MRSA, n (%)	1 (1.0)
MDRO colonization during follow-up, n (%)	31 (32.0)
ESBL E. coli, n (%)	3 (3.1)
ESBL K. pneumoniae, n (%)	2 (2.1)
CR Enterbacteriaceae, n (%)	0 (0)
Amp-C Enterobacter spp., n (%)	4 (4.1)
CR P. aeruginosa spp., n (%)	1 (1.0)
VR Enterococcus spp., n (%)	22 (22.7)
other MDR bacteria, n (%)	3 (3.1)
MRSA, n (%)	0 (0)
MDRO infection during follow up, n (%)	13 (13.4)
ESBL E. coli, n (%)	4 (4.1)
ESBL K. pneumoniae, n (%)	1 (1.0)
CR Enterbacteriaceae, n (%)	0 (0)
Amp-C Enterobacter spp., n (%)	3 (3.1)
CR P. aeruginosa spp., n (%)	1 (1.0)
VR Enterococcus spp., n (%)	6 (6.2)
Other MDR bacteria, n (%)	2 (2.1)
MRSA, n (%)	0 (0.0)
Infections with the same MDRO isolated in rectal swaps	11 (84.6)

Abbreviations: CR, carbapenem-resistant; ESBL, Extended-spectrum beta-lactamase; MDRO, multidrug-resistant organisms; MRSA, methicillin-resistant *Staphylococcus aureus*; VR, vancomycin-resistant.
