# Supplementary material for: Impact of colonization with multidrug-resistant organisms on antibiotic prophylaxis in patients with cirrhosis and variceal bleeding
Source: PLoS One. 2022 May 24;17(5):e0268638. doi: 10.1371/journal.pone.0268638 (PMC9128949; doi:10.1371/journal.pone.0268638)
Supplement: S3 Table — (PDF) [file pone.0268638.s003.pdf]

S3 Table. Patients' characteristics according to the adequacy of antibiotic prophylaxis.

Characteristics	**Adequate antibiotic prophylaxis (n=68)**	**Inadequate antibiotic prophylaxis (n=29)**	**P-value**
Age, y, median (IQR)	58 (12)	55 (7)	0.991
Male sex, n (%)	54 (79.4)	22 (75.9)	0.789
Etiology of cirrhosis			
Alcohol, n (%)	31 (45.6)	17 (58.6)	0.273
Viral Hepatitis, n (%)	18 (26.4)	6 (20.7)	0.616
NASH, n (%)	3 (4.4)	1 (3.4)	1.000
Cryptogenic, n (%)	4 (5.9)	1 (3.4)	1.000
other, n (%)	12 (17.6)	4 (13.8)	0.770
ICU admission during hospital stay, n (%)	66 (97.1)	27 (93.1)	0.581
Days on ICU, median (IQR)	5(3)	4(2)	0.574
Hemorrhagic shock at bleeding, n (%)	42 (61.8)	12 (41.4)	0.077
Stage of liver disease			
MELD-Score, median (IQR)	17 (5)	17 (6)	0.270
Child-Pugh B/C n (%)	59 (86.7)	23 (79.3)	0.370
Ascites, n (%)	50 (73.5)	21 (72.4)	1.000
ACLF present at bleeding, n (%)	34 (50.0)	12 (41.4)	0.508
Endoscopy findings/therapy			
Time to endoscopy, hours, IQR	2 (1)	2 (1)	1.000
Grade of EV	2 (0)	2 (0)	1.000
Active bleeding at endoscopy	45 (66.2)	21 (72.4)	0.638
Additional fundus varices	24 (35.3)	7 (24.1)	0.346
Endoscopic treatment	63 (92.6)	28 (96.6)	0.665
EV ligature	48 (70.6)	23 (79.3)	0.458
Injection therapy	14 (20.6)	5 (17.2)	0.787
EV Stenting	2 (2.9)	1 (3.4)	1.000
TIPS placement during hospitalization	7 (10.3)	4 (13.8)	0.729
Terlipressin treatment for 3-5 days	54 (79.4)	23 (79.3)	1.000
MDRO colonization at baseline, n (%)	9 (13.2)	25 (86.2)	**< 0.0001**
Outcome, n (%)			
ACLF day 7	18 (26.5)	8 (27.6)	1.000
Liver transplantation	5 (7.4)	1 (3.4)	0.665
Death/liver transplantation within			
30 days, n (%)	23 (33.8)	5 (17.2)	0.142
365 days, n (%)	29 (59.2)	14 (48.3)	0.659

Abbreviations: ACLF, acute-on-chronic liver failure; CI, confidence interval; EV, esophageal varices; ICU, intensive care; IQR; interquartile-range; MELD, model for end-stage liver disease; MDRO, multidrug-resistant organism; NASH, non-alcoholic steato-hepatitis; SBP, spontaneous bacterial peritonitis.
